# Short-term impact of different doses of spent coffee grounds, salt, and sand on soil chemical and hydrological properties in an urban soil

**DOI:** 10.1007/s11356-023-28386-z

**Published:** 2023-07-04

**Authors:** Muhammad Owais Khan, Anna Klamerus-Iwan, Dawid Kupka, Ewa Słowik-Opoka

**Affiliations:** 1 Department of Ecological Engineering and Forest Hydrology, University of Agriculture in Krakow, Krakow, Poland; 2 Department of Forest Ecology and Sylviculture, University of Agriculture in Krakow, Krakow, Poland

**Keywords:** Total carbon, Carbon dioxide emission, *WDPT*, Volumetric water content, Water storage capacity

## Abstract

Natural and human activities have deteriorated urban soil’s health and ecological functions as compared to forest soils. Therefore, we hypothesized that any intervention in poor quality soil in urban area will change their chemical and water retention properties. The experiment was conducted in Krakow (Poland) in completely randomized design (CRD). The soil amendments used in this experiment consisted of control, spent coffee grounds (SCGs), salt, and sand (1 and 2 t ha^−1^) in order to evaluate the impact of these soil amendments on the urban soil chemical and hydrological properties. Soil samples were collected after 3 months of soil application. The soil pH, soil acidity (me/100 g), electrical conductivity (mS/cm), total carbon (%), CO_2_ emission (g m^−2^ day^−1^), and total nitrogen (%) were measured in laboratory condition. The soil hydrological properties like volumetric water content (*VWC*), water drop penetration time (*WDPT*), current water storage capacity (*S*_a_), water storage capacity after 4 and 24 h (*S*_4_ and *S*_24_), and capillary water *P*_k_ (mm) were also determined. We noted variations in soil chemical and water retention properties in urban soil after the application of SCGs, sand, and salt. It was observed that SCGs (2 t ha^−1^) has reduced soil pH and nitrogen (%) by 14 and 9%, while the incorporation of salt resulted in maximum soil EC, total acidity, and soil pH. The soil carbon (%) and CO_2_ emission (g m^−2^ day^−1^) were enhanced and declined by SCGs amendment. Furthermore, the soil hydrological properties were significantly influenced by the soil amendment (spent coffee grounds, salt, and sand) application. Our results showed that spent coffee grounds mixing in urban soil has considerably enhanced the soil *VWC*, *S*_a_, *S*_4_, *S*_24_, and *P*_k_, whereas it decreased the water drop penetration time. The analysis showed that the single dose of soil amendments had not improved soil chemical properties very well. Therefore, it is suggested that SCGs should be applied more than single dose. This is a good direction to look for ways to improve the retention properties of urban soil and you can consider combining SCGs with other organic materials like compost, farmyard manure, or biochar.

## Introduction

Soils play a crucial role in the Earth’s system, and they are vital in achieving many of the UN Sustainable Development Goals (Keesstra et al. 2016a). According to the United Nations, soil protection is a key land-use policy issue, and strategies are needed to maintain soil quality, soil functions, and soil services for sustainability (Keesstra et al. 2018). Urban soils play an essential role in urban ecosystems, providing a growth medium for plants, vegetation, and soil microorganisms (Guilland et al. 2018). Soil water retention, fertility maintenance, and contaminant removal are services urban soils provide (Ozdemir 2019; Salmond et al. 2016). However, compared to natural forest soils, human activities have aggravated urban soils’ health and ecological function (Weissert et al. 2016; Zhao et al. 2013). As a result, growing plants in this soil is typically complex, and maintaining green land is also costly (Zou et al. 2012; Miao and Shi 2015). Climate change and its consequences, such as drought, have recently become more severe and pervasive worldwide, particularly in arid and semi-arid areas (Solomon et al. 2007). Precipitation is becoming more unpredictable, average temperatures are rising, and soil and water resources are deteriorating daily (Knox et al. 2012). Furthermore, an extreme reduction in rainfall due to global warming has been shown to enhance the severity and frequency of urban droughts, posing a severe danger to the whole ecological services provided by urban green zones (Gillner et al. 2014; Mullaney et al. 2015). These issues could decrease the urban soil water retention properties and destroy various species, from yearly flowering grasses to perennial crossroad trees, resulting in massive economic and ecological losses.

Soil degradation caused by climate change and human activities has resulted in the deterioration of soil health worldwide, with effects such as soil erosion, nutrient depletion, organic matter reductions, and compaction (Olsson et al. 2019). Urban trees are hampered by several environmental factors that limit their growth and shorten their lives (Nilsson et al.  2001). Chloride salts are extensively used in cold weather cities to manage ice and snow on roadways and pathways throughout the wintertime. Transportation organizations use sodium chloride (NaCl) most often because of its availability, efficiency, and low cost (Transportation Association of Canada 2004). It has already been demonstrated that salt application for winter road maintenance increases soil salinity (Fay and Shi 2012). Urban trees can take up the accumulated salt from the soil during the growing period (Cunningham et al. 2008). As salt flow is highest near roadways, trees near salt-treated areas are most affected by salt stress (Cekstere and Osvalde 2013). Whereas de-icing salt is identified as a leading cause in the decrease of urban trees, its use has expanded over the past decade as a result of growing public demand for safe driving and better road traffic (Fay and Shi 2012). As a result, there is a broader societal interest in improving soil quality by adopting sustainable soil management techniques that improve soil properties, especially organic matter content (caused by grass mowing and leaf raking for many years), and so assist to develop healthy soil. Therefore, developing novel methods for enhancing urban soil quality and water retention capacity is valuable and significant.

rom the perspective of climate change, the knowledge of carbon sequestration and water retention in all types of ecosystems has gained significance as it may assist in the mitigation of and adaptation to them (Prasad and Pietrzykowski 2020). The availability of plant-available green water in the soil must be improved through the application of solutions based on nature (nature-based solutions), as well as controlling the amount and quality of blue water (surface water and groundwater) throughout the year(s) to prevent floods and droughts (Keesstra et al. 2021). In droughts, heat waves, and storms, dry soil becomes hydrophobic and less permeable, and in the event of heavy rains, it can contribute to local flood episodes (Zscheischler et al. 2018). By 2050, the world population is expected to grow between 8 and 11 billion people, with 66 percent of people living in cities (UNDESA 2014). Two critical difficulties for a highly urbanized world’s population are providing essential resources (food, water, and power) to urban centers and the management of urban wastes produced in urban centers. New, inventive, and sustainable urban solutions are necessary to tackle these difficulties (Hoornweg and Bhada-Tata 2012). Soil amendments could be utilized in urban horticulture or food production, as well as soil remediation, as an example of how organic food waste created in cities could be exploited (Brown et al. 2011) and to enhance city sustainability and ecological impacts (Martinez-Blanco et al. 2009). It is not a new concept to use organic food waste as a soil additive; it has been used as a soil amendment by civilizations throughout history (Parr and Hornick 1992). Organic soil fertilizers can help crop growth by improving important physicochemical and biological characteristics (Brady and Weil 2008). 2014 Organic waste amendment has improved the nutrient availability, soil moisture, nutrient-holding capabilities, soil structure and water infiltration, soil pH, reduced nitrate leaching, soil biological characteristics, and long-term carbon sequestration (Haider et al. 2014).

A large quantity of spent coffee grounds (SCGs) is produced all over the world each year (15 million tons) (Kamil et al. 2019). According to Stylianou et al. (2018), SCG can be used as an organic soil amendment and has been proven to have many environmental benefits. Even though SCG is phytotoxic, it has been shown that it can improve soil physical and chemical fertility, and that it can affect the soil microbiota as well. SCG has a nitrogen level of 1.0 to 2.5 percent and a C/N ratio of 20 to 25, making it much higher than typical horticulture soils and soil microbial communities (Pujol et al. 2013). Researchers have evaluated the impact of SCGs on soil physical, chemical, and biological properties on Mediterranean soils (Cervera-Mata et al., 2021, 2022; Comino et al. 2020). They reported that the application of SCGs enhances water retention; total porosity; and N, P, and K concentrations and improves C cycle, while reduces the bulk density of the soil. Cervera-Mata et al. (2018) concluded that the usage of SCGs in soils enhances the SOC and reduces the emissions of CO_2_ to the environment. The phenols in SCG are toxic to soil microbes and plants, but they also act as natural pesticides and herbicides (Cruz et al. 2012). Morikawa and Saigusa (2008) found that composted coffee grounds improved the growth of various horticultural crops in specified soils, while the results for non-composted SCG are less apparent. Although the results vary depending on the plant species, soil amendment with SCG can simultaneously increase plant biomass while lowering foliar N content (Yamane et al. 2014; Cruz et al. 2012). Since spent coffee grounds are acidic, it may lower soil pH (Mussatto et al. 2011).

Interdisciplinary exploration and understanding of the functioning of the urban greenery ecosystem in the changing abiotic conditions are necessary for modeling hydrological processes and the carbon cycle. We hypothesized that the addition of SCGs in poor quality soil in cities will not only change soil chemical properties, but will also improve the soil hydrological properties due to organic nature; nevertheless, a single dose of SCGs, salt, and sand is a determinant of the direction for further research. We designed this experiment in order to study how different doses of SCGs as an organic amendment source could improve the soil chemical and hydrological properties related to volumetric water content, water storage capacity, and capillary water, as compared to salt application in urban soil.

The results of the conducted research should be considered in a long-term perspective and are expected to broaden the knowledge both in the field of biological sciences and facilitate the verification of research methods in the field of hydrological sciences and environmental engineering.

## Materials and methods

### Description of experimental site and soil sampling

**Fig. 1:**
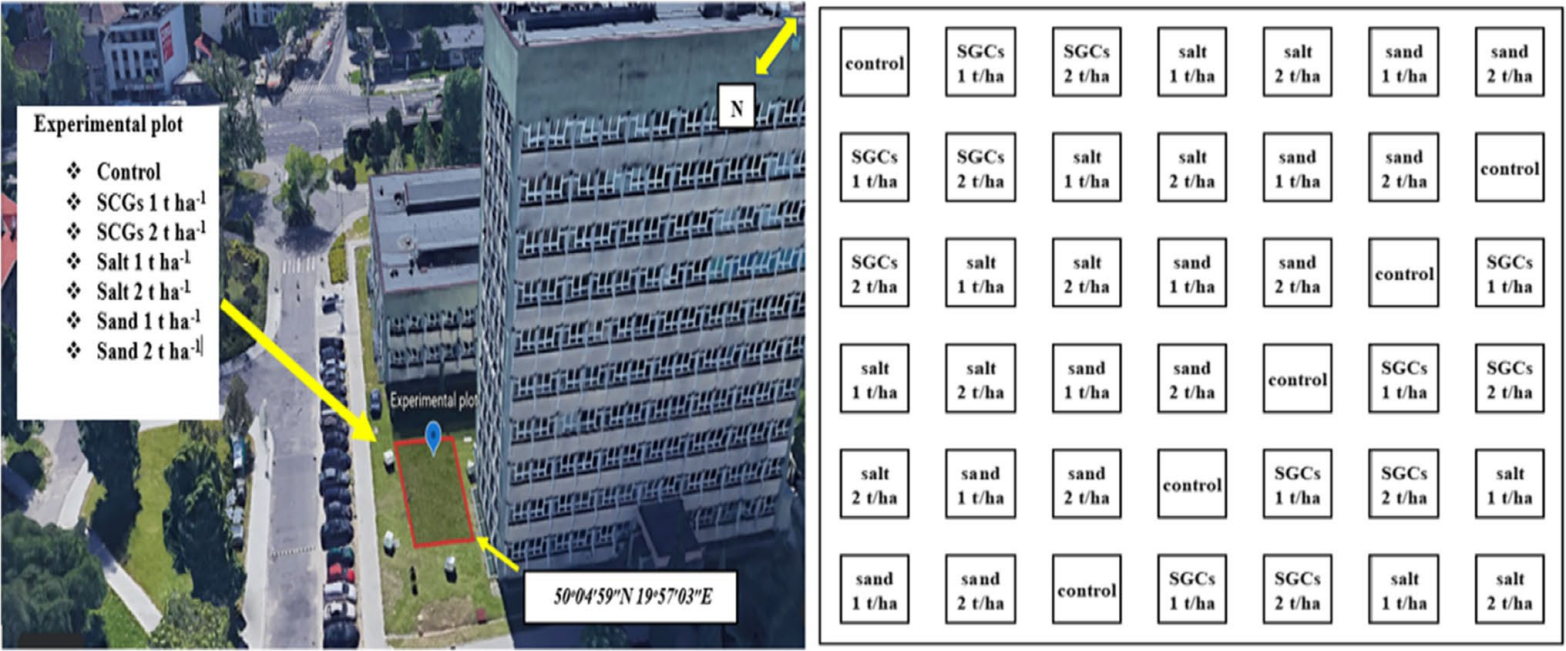
Location of the study site and experimental layout

The experiment was conducted in winter season (2022) at the University of Agriculture in Krakow Campus, which is located in the urban area of the Krakow City. The study site was established in less frequented lawn, and before conducting experiment, the area was cleaned from tree’s branches, leaves, and other unwanted material (Fig. 1). A moderately cold climate prevails in the city of Krakow from January to March, average rainfall was about 50 mm for the examined months, and the average temperature ranged from − 2 to 3 °C. The average relative humidity of the air recorded at that time was 79%. The soil in the study plot was identified as Urbic Technosol according to the World Reference Base for Soil Resources (IUSS Working Group WRB-FAO 2015). The soils were formed on Quaternary sands, which are one of the main (along with loess and alluvium) parent materials within the city of Krakow.

The experiment was conducted in completely randomized design (CRD) and the individual plot area was 1 × 1 m^2^. The treatments used in this experiment were spent coffee grounds (SCGs), salt, sand, and control (no soil amendment). Each treatment was applied in two levels consisting (1 and 2 t ha^−1^) with one control plot. Each experimental unit was replicated six times. The soil samples were collected (10-cm depth) by using 100-cm^3^ Kopecky cylinders after 3 months of treatment in order to assess the chemical and hydrological properties.

The soil amendment SCGs were collected from the different coffee shops. The SCGs were acidic in nature, the nitrogen content was 0.8–2.3%, and the C/N ratio was 18 to 22. The salt used in this study sodium chloride (NaCl) was collected from the “Kłodawa Salt Mine” region. The NaCl was 90% pure, and up to 8% the content of substances was insoluble in water. The potassium ferrocyanide (K_4_(Fe (CN)_6_) 20 mg kg^−1^ was also added to the NaCl, which is an anti-caking agent. The sand utilized in this experiment was collected from the aggregate mine “Kruszywo” Krakow. The mineralogical composition of sand was silica (SiO_2_) and the size was “fine” size ranged from 0 to 0.5 mm grade II.

### Hydrological properities

Soil samples were collected from each experimental plot and we weighed fresh (*M*_f_) and then immersed in distilled water under room conditions (± 21 °C, humidity 30%). The samples were weighed 4 h (*M*_4_) after the cylinders were completely filled with water, and then after 24 h (*M*_24_) by adding the time the samples were out of water when weighing after 4 h. Then the samples were dried in a laboratory drier for another 24 h at 105 °C, obtaining a dry mass (*M*_d_).

The current water storage capacity (*S*_a_) was obtained by subtracting *M*_d_ from *M*_f_ and then *S*_4_ was obtained by subtracting *M*_4_ from *M*_d_ and *S*_24_ by subtracting *M*_24_ from *M*_d_ (Klamerus-Iwan et al. 2020). The water drop penetration time (*WDPT*) test determines how long it takes for a single water drop to enter a sample of soil (Doerr 1998; Hallin et al. 2013). A medical dropper was used to placed three to five drops of distilled water of a similar volume to the surface of each sample, and the duration it took for each drop to fully enter the soil was timed using a stopwatch. Using the drop penetration time measurement data for each soil samples, the average value (*WDPT*_av_) and median (*WDPT*_me_) were computed for further study. The *WDPT* measurement was performed in 2 variants: on a fresh sample (*WDPT*_1) and on samples taken in steel frames (20 × 20 × 20) and placed in laboratory conditions for 5 days (*WDPT*_2). This method allowed us to observe the reaction to drying of samples with additions of SCGs, salt, and sand to the soil.

The volumetric water content (*VWC*%) in the soil was measured by TEROS_12 (Meter n.d.). TEROS_12 sensor monitors the dielectric permittivity of the surface layer using an electromagnetic field. The sensor uses a 70-MHz oscillating wave to the sensor needles, which charge in line with the material’s dielectric. The charge time is linked to the dielectric constant and *VWC* of the substrate. Microprocessor TEROS 12 measures the charging time and outputs a raw value based on the dielectric permittivity of the substrate. The raw data is then transformed to *VWC* using a substrate-specific calibration equation. *VWC* (*θ*) is given by the following equation:

$$\theta \left({\mathrm{m}}^{3}/{\mathrm{m}}^{3}\right)=3.879\times {10}^{-4}\times RAW-0.695$$

The *VWC* measurement was performed in 2 variants: on a fresh sample (*VWC*_1) and on samples taken in steel frames and placed in laboratory conditions for 5 days (*VWC*_2).

A soil medium’s water reservoir’s maximum capacity is defined by its capillary capacity, which is calculated over long periods of time and under maximum storage conditions. In order to measure the capillary capacity (*P*_k_), individual monoliths were soaked in water for 7 to 10 days, with their initial soaking of 2 to 3 days consisting of gradual filling with water (Ilek et al. 2017). The water in the containers was replaced every 2 days to avoid decay. The capillary capacity *P*_k_ (mm) was determined according to the following formula:

$${P}_{\mathrm{k}}=\left(v/V\right)\times 10$$

In this case, *v* represents the volume of water (cm^3^) calculated by subtracting the difference between the mass of a given soil horizon, when it is at maximum water storage capacity, from the mass of that soil horizon, after drying to 105 °C. In the state of maximum water storage capacity (cm^3^), *V* represents the volume of a given horizon.

The granulometric composition was determined by laser diffraction, divided into sand, dust, and clay. Laser Particle Sizer ANALYSETTE 22 was used to perform this division (Fritsch 2016).

### Chemical analysis

Also, soil samples were collected from each experimental unit in plastic tube of 100-cm^3^ volume. The soil samples were air dried; removed stones, roots, leaves, and other unwanted material; and then sieved through a 2-mm sieve for chemical analysis. Soil samples thus ready, 10 g was taken from each treatment and grounded in a ball mill (Fritsch) for the determination of nitrogen (N%) and carbon (C%) concentrations in a LECO TrueMac Analyser (Leco, St. Joseph, MI, USA). A potentiometric method using a combined electrode and soil suspension in distilled water (1:5 mass-to-volume ratio) was used to measure soil pH after 24 h of equilibration (Buurman et al. 1996). In order to determine the total acidity (TA), 10 g of soil was extracted with 1 M calcium acetate ((CH_3_COO)_2_Ca), shaken for 1 h, and filtered. The samples on filters were washed with 100 mL of extractant solution. Twenty-five milliliters of the obtained extract was titrated to pH 8.2 with 0.1 M NaOH using potentiometric titration (automated titrator Mettler Toledo) (Buurman et al. 1996).

### Measurement of soil CO_2_ respiration

A closed chamber incubation method with sodium hydroxide (NaOH) was used to evaluate soil carbon dioxide emissions (Hopkins 2006). We poured 30 mL of 1 M NaOH into a beaker and applied it to each soil column. In accordance with Eq. (1), CO_2_ emission from the soil was converted into Na_2_CO_3_:

1 $$2\mathrm{NaOH}+{\mathrm{CO}}_{2}\to {\mathrm{Na}}_{2}{\mathrm{CO}}_{3}+{\mathrm{H}}_{2}\mathrm{O}$$

A barium chloride solution was not needed to precipitate carbonates because the soil samples were free of carbonates. The soil columns with beakers were placed in an airtight plastic bag to make sure that the soil moisture remains unchanged and the proper measurement of CO_2_ emission, and then stored in an incubator at 20 °C. Following a week of incubation, the sodium hydroxide excess was backtitrated using 0.5 M HCl through potentiometric titration (Automatic titrator, Mettler Toledo, Inc. Columbus, OH). According to Eq. (2), the backtitration was carried out.

2 $$\mathrm{NaOH}+\mathrm{HCI}\to \mathrm{NaCl}+{\mathrm{H}}_{2}\mathrm{O}$$

The amount of carbon dioxide emission was depicted in g_CO2_ m^−2^ day^−1^.

### Statistical analysis

The analysis of variance (ANOVA) was done for the collected data using MS Excel, and the LSD (least significant difference) tests for the significant differences between treatments (control, SCGs, salt, and sand at the rate of 1 and 2 t ha^−1^) were performed through Statistic software (Statistix 8.1). Tukey (HSD) test was done through Python software to test differences among treatment means for significance. The boxplots were created through python using seaborn library (Waskom et al. 2020). The principal component analysis (PCA) and the regression plots were done through R statistical software using packages “Factoextra,” “FactoMiner,” and “ggplot2” (Kassambara 2017; Le et al. 2008; Wickham 2016). The significance level 95% (*p* < 0.05) was tested in this experiment.

## Results

The tests showed that all samples contained a total of 49.2% sand, 45% dust, and 5.8% clay. The results, based on the PTG 2008 standards, show that the area from which the samples were taken is composed mainly of clay formations, a subgroup of sandy loams.

The characteristic properties of this type of soil formations are that they do not dry out too quickly, providing plants with constant access to water, and their roots, access to oxygen, due to the fact that they are properly aerated, but their properties do not allow excess water to accumulate.

### Soil chemical properties

**Fig. 2:**
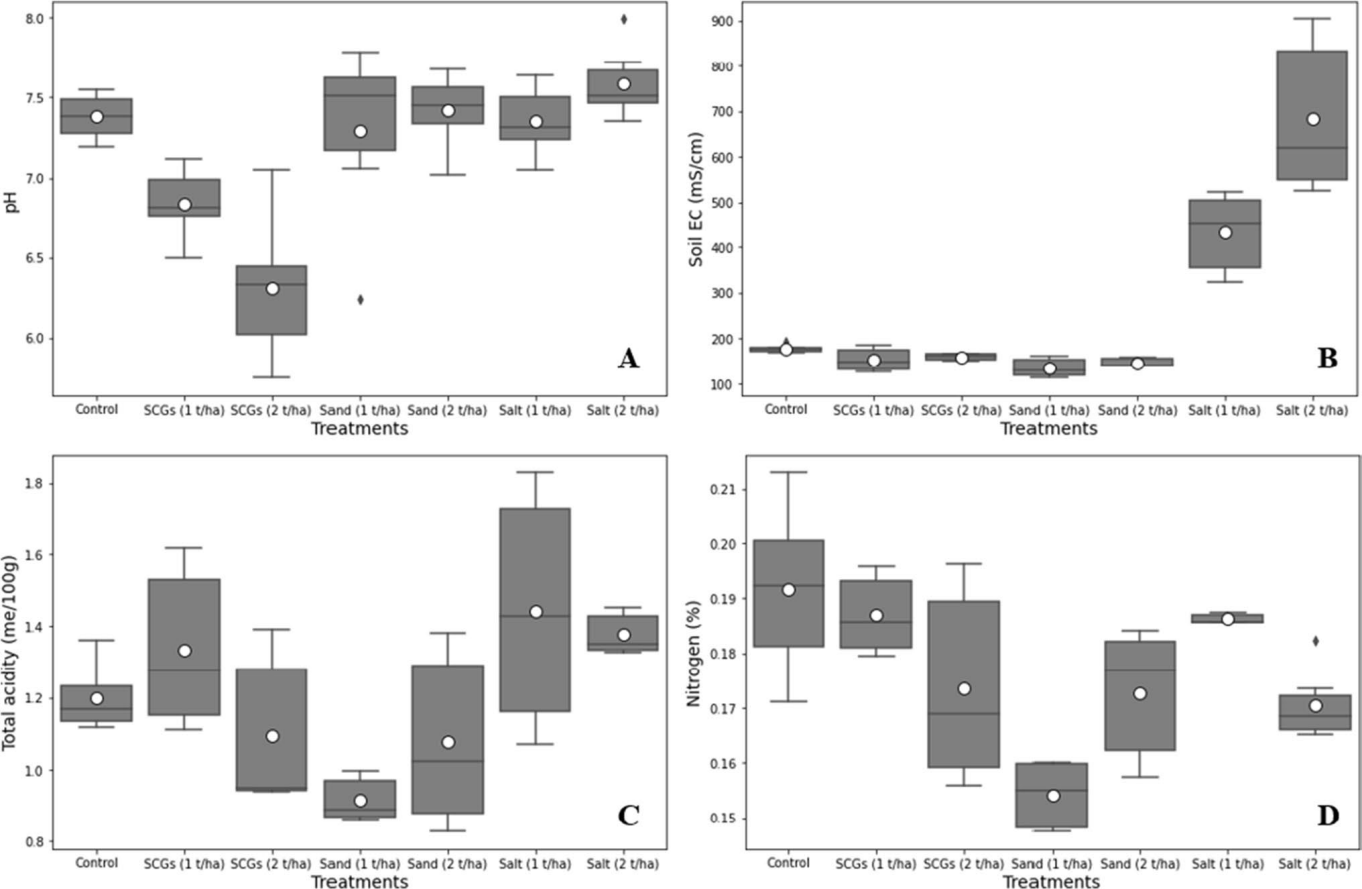
**A**, **B**, **C**, and **D** Boxplot of the soil pH, EC (mS/cm), total acidity (me/100 g), and nitrogen (%) plotted against different treatments (control, SCGs, sand, and salt at the rate of 1 and 2 t ha^−1^). The upper and lower whiskers represented the highest and lowest values, the middle line within the boxplot represents the median value, and the white circle inside the boxplot shows the mean value of each treatment

The mixing of different doses of SCGs, salt, and sand significantly influenced the soil pH, electrical conductivity (EC), total acidity, and the soil nitrogen (%). The clear differences in soil pH with different doses of soil amendments can be seen in Fig. 2 A, where SCG doses (1 and 2 t ha^−1^) have a significant affect on soil pH rather than sand and salt doses. Soil pH was more alkaline when treated with salt (2 t ha^−1^) and the application of SCGs (2 t ha^−1^) decreased the soil pH as compared to control plot. Additionally, a decrease in soil pH by 7 and 14% was recorded with the application of SCGs at doses of 1 and 2 t ha^−1^. At 1 and 2 t ha^−1^ of salt, there was a noticeable rise in the electrical conductivity of the soil. However, other amendments, such as SCGs or sand at 1 and 2 t ha^−1^, did not significantly affect soil’s electrical conductivity (Fig. 2 B). Furthermore, compared to the control, salt addition raised soil EC by 143 and 283 percent, respectively. Soil EC was the lowest in the plot with 1 t ha^−1^ of sand. The statistical results indicated that the total acidity significantly varied after the incorporation of different treatments (SCGs, sand, and salt); however, their different levels (1 and 2 t ha^−1^) results were not quite different from each other. The highest TA value was recorded in salt, followed by SCGs and sand. Furthermore, salt at the rate of 1 and 2 t ha^−1^ increased total acidity by 20 and 15 percent compared with control treatment (Fig. 2 C). The nitrogen concentrations in the soil after incorporation of different amendments, i.e., SCGs, salt, and sand at the rate of 1 and 2 t ha^−1^, varied significantly from each other. The highest nitrogen concentration was recorded in the control plot, followed by SCGs at the level of 1 t ha^−1^; however, the minimum nitrogen concentration was measured in the sand treatment at the rate of 1 t ha^−1^. In addition, the mixing of SCGs at the rate of 1 and 2 t ha^−1^ significantly declined the nitrogen contents by 2 and 9 percent as compared to the control (Fig. 2 D).

### Carbon (%) and CO_2_ emission

**Fig. 3:**
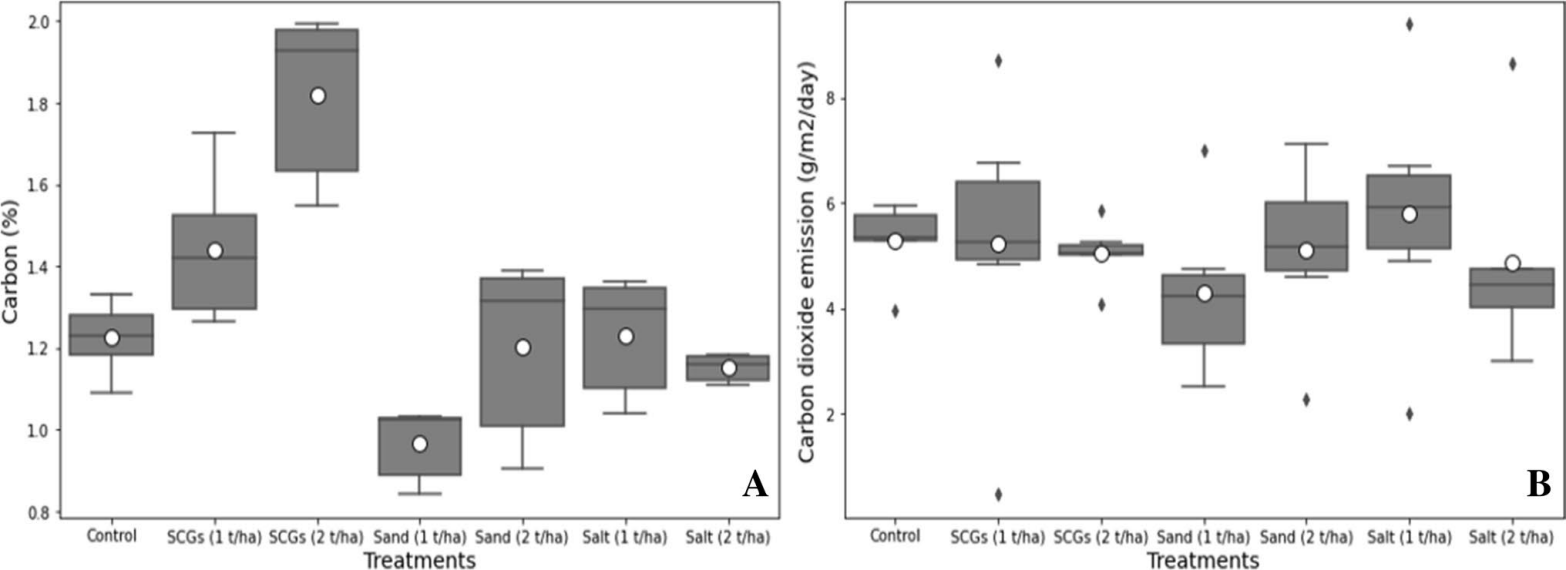
**A** and **B** Boxplot of the soil carbon (%) and CO_2_ emission (g/m^2^/day) plotted against different treatments (control, SCGs, sand, and salt at the rate of 1 and 2 t ha^−1^). The upper and lower whiskers represented highest and lowest values, the middle line within the boxplot represents the median value, and the white circle inside the boxplot shows the mean value of each treatment

The impact of different amendments significantly influenced the carbon (%) concentration in the soil, while the CO_2_ emission was not significantly affected. The maximum carbon (%) content was noted in the plot, which was treated with SCGs at the rate of 2 t ha^−1^ followed by SCGs (1 t ha^−1^) (Fig. 3 A). The minimum carbon concentration was recorded in the sand (1 t ha^−1^) treatment. Furthermore, the maximum CO_2_ emission was noted in the salt treatment (1 t ha^−1^), and the SCG (2 t ha^−1^) has considerably reduced the CO_2_ emission from the soil (Fig. 3 B).

### Soil hydrological properties

#### Volumetric water content

**Fig. 4:**
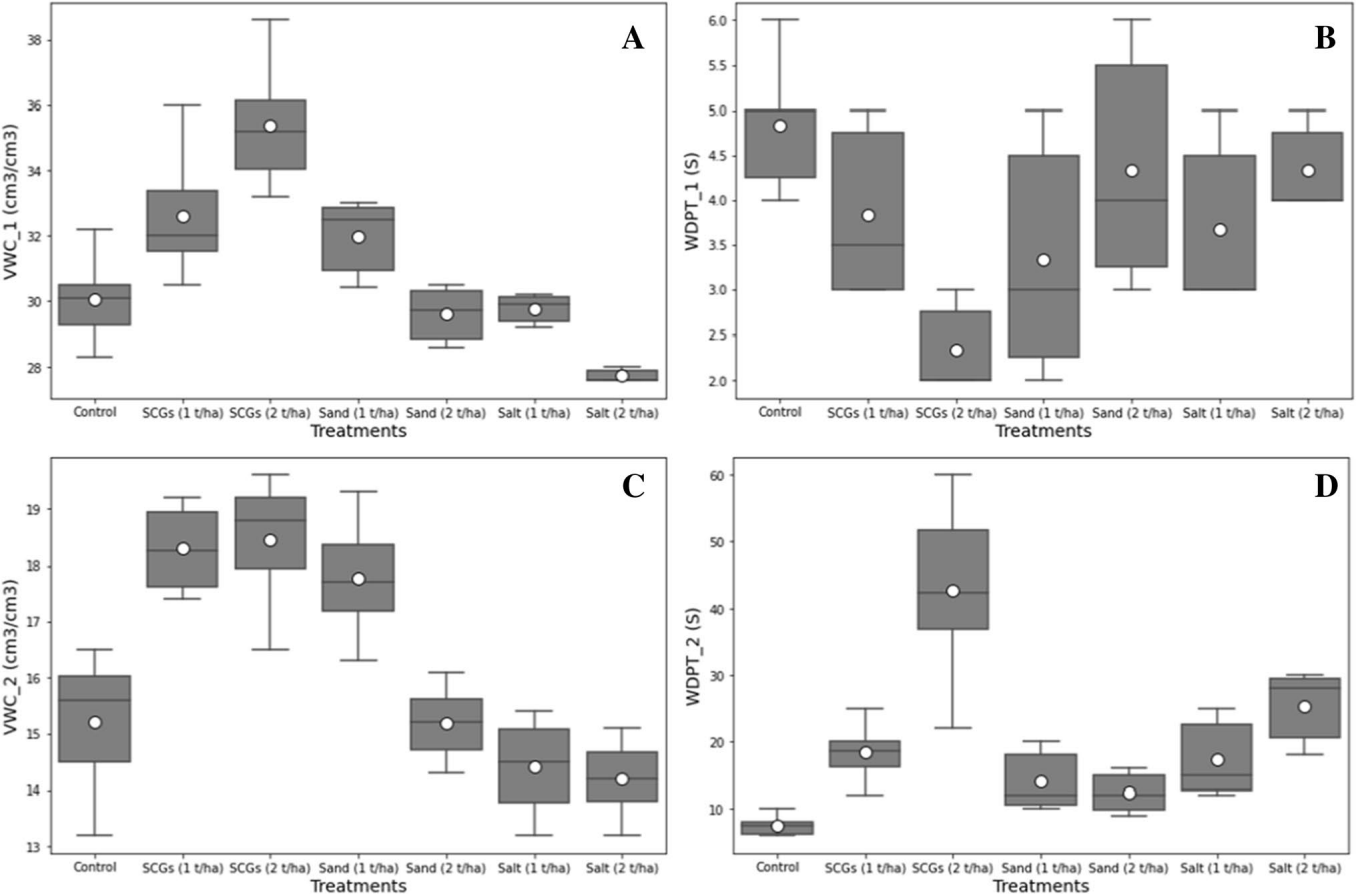
**A**, **B**, **C**, and **D** Boxplot of the volumetric water content (*VWC*_1), water drop penetration time (*WDPT*_1), volumetric water content in soil after 5 days (*VWC*_2), and water drop penetration time (*WDPT*_2) plotted against different treatments (control, SCGs, sand, and salt at the rate of 1 and 2 t ha^−1^). The upper and lower whiskers represented highest and lowest values, the middle line within the boxplot represents the median value, and the white circle inside the boxplot shows the mean value of each treatment

The impact of the different soil amendments depicted significant differences in the mean value of the soil volumetric water content in the fresh soil samples. The volumetric water content of the soil in the fresh state (*VWC*_1) has been dramatically decreased by the addition of salt (1 and 2 t ha^−1^). In comparison, the application of SCGs (1 and 2 t ha^−1^) has consistently improved the volumetric water content in the field by 9 and 18%, compared with control, respectively. The SCG (2 t ha^−1^) treatment had the greatest computed *VWC*_1, whereas the salt (2 t ha^−1^) treatment had the lowest *VWC*_1 (Fig. 4 A).

The integration of several soil treatments significantly influenced the data of volumetric water content in the soil samples after 5 days in the lab (*VWC*_2) (Fig. 4 C). The SCGs treatment (2 t ha^−1^) has escalated (21%) *VWC*_2, followed by SCGs (1 t ha^−1^) up to 20% in soil samples after 5 days at room temperature. Furthermore, the *VWC*_2 concentration plunged in other treatments after 5 days of drying at room temperature.

#### Water drop penetration time (*WDPT*_1 and *WDPT*_2)

The statistical analysis of the water drop penetration time data in the fresh soil samples (*WDPT*_1) showed significant variations among the application of different soil amendments. The least water drop penetration time (2.33 s) was taken in the treatment of SCGs at the rate of 2 t ha^−1^, while the control plot took the maximum time (4.83 s) for the water drop to penetrate (Fig. 4 B). There was no considerable differences between SCGs (1 t ha^−1^), sand (2 t ha^−1^), and salt (2 t ha^−1^) treatments. The incorporation of various soil treatments has considerably differentiated the *WDPT*_2. The highest water drop penetration time (42.8 s) was recorded in SCGs (2 t ha^−1^) treatment, while the least time (7.5 s) for water drop penetration was taken in the control plot treatment (Fig. 4 D). Further, the impact of sand treatment at both doses (1 and 2 t ha^−1^) showed no significant effect on *WDPT*_2.

#### Current water storage capacity (*S*_a_), water storage capacity after 4 h (*S*_4_), maximum water capacity after 24 h (*S*_24_), and capillary water in the 1-cm layer of soil

**Fig. 5:**
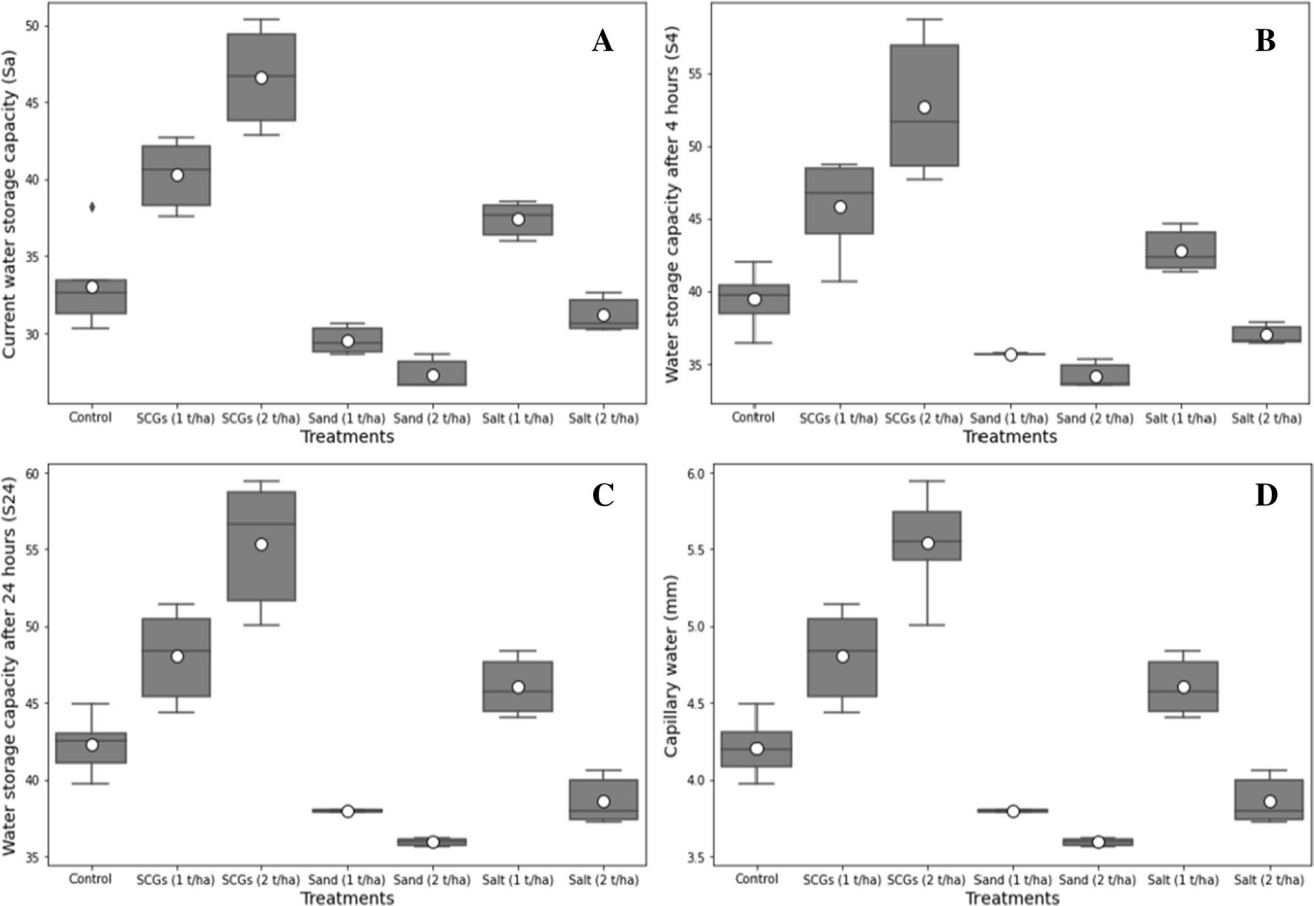
**A**, **B**, **C**, and **D** Boxplot of the current water storage capacity (*S*_a_), water capacity after 4 h (*S*_4_), water capacity after 24 h (*S*_24_), and capillary water *P*_k_ (mm) plotted against different treatments (control, SCGs, sand, and salt at rate of 1 and 2 t ha^−1^). The upper and lower whiskers represented highest and lowest values, the middle line within the boxplot represents the median value, and the white circle inside the boxplot shows the mean value of each treatment

The *S*_a_, *S*_4_, and *S*_24_ have been significantly impacted by the effects of various treatments, including SCGs, salt, and sand. Compared to other treatments, the application of SCGs (2 t ha^−1^) improved these parameters. In contrast, the *S*_a_, *S*_4_, and *S*_24_ in soil samples have been dramatically reduced by the addition of sand (2 t ha^−1^). In addition, the SCG (2 t ha^−1^) has increased the *S*_a_, *S*_4_, and *S*_24_ by 71, 54, and 54%, compared with the declining factor (sand, 2 t ha^−1^), respectively (Fig. 5 A, B, C). The capillary water in the 1-cm layer of the soil was increased notably after incorporation of the soil treatment SCGs at the rate of 1 and 2 t ha^−1^ as compared to other amendments (Fig. 5 D). However, the application of sand and salt showed minimum capillary water in the soil layer as compared to the SCGs and control plot. Moreover, the SCGs treatment at the rate of 1 and 2 t ha^−1^ escalates the capillary water up to 14 and 32%, compared with control, respectively.

#### Linear correlation between *S*_a_ and *S*_4_, *S*_a_ and *S*_24_, *S*_a_ and capillary water *P*_k_ (mm), carbon (%) and *WDPT*_1 (S), and carbon (%) and *WDPT*_2 (S)

**Fig. 6:**
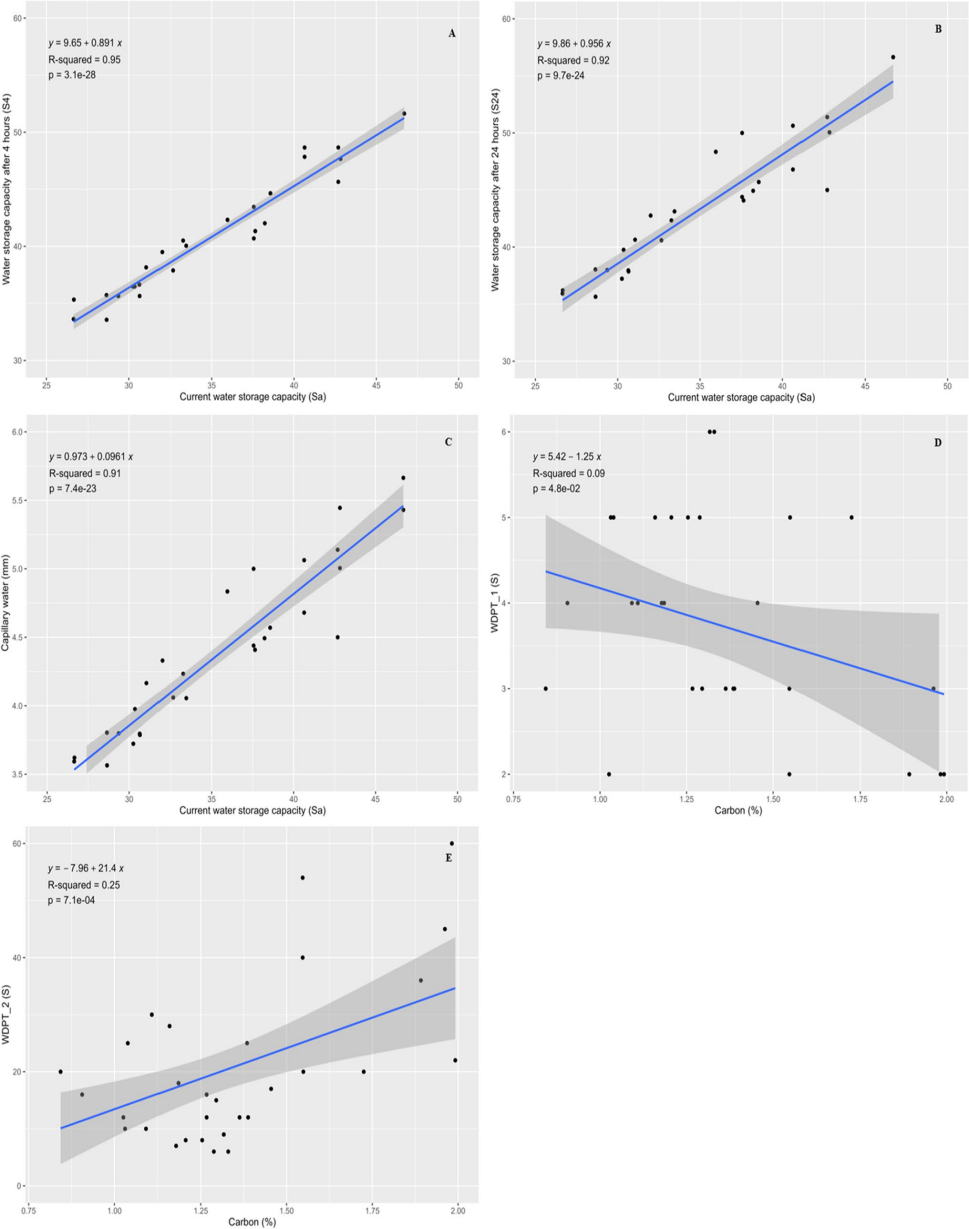
**A**, **B**, **C**, **D**, and **E** Linear regression between current water storage capacity (*S*_a_) and water storage capacity after 4 h (*S*_4_), current water storage capacity (*S*_a_) and water storage capacity after 24 h (*S*_24_), current water storage capacity (*S*_a_) and capillary water *P*_k_ (mm), carbon (%) and *WDPT*_1 (S), and carbon (%) and *WDPT*_2 (S)

Figure 6 shows the correlation between current water storage capacity (*S*_a_) and water capacity after 4 h (*S*_4_), *S*_a_ and maximum water storage capacity after 24 h (*S*_24_), *S*_a_ and capillary water (mm), carbon (%) and water drop penetration time (*WDPT*_1), and carbon (%) and water drop penetration time (*WDPT*_2). Figure 6 A depicts the correlation among current water storage capacity (*S*_a_) and water capacity after 4 h (*S*_4_). The *R* squared value is higher, which explains that the data is well fitted in this model. Further, the coefficient value indicates a positive relationship between dependent and independent variables. Finally, the *p* value shows statistically significant relationship between *S*_a_ and *S*_4_. The linear regression between *S*_a_ and *S*_24_ is presented in Fig. 6 B, which indicates that increasing in the current water storage capacity also tend to increase the water storage capacity after 24 h in soil sample. The coefficient represents the positive relation among *S*_a_ and *S*_24_, which means that water storage capacity after 24 h increases by increasing in current water storage capacity, and the *p* value (< 0.05) indicates the statistical significance among *S*_a_ and *S*_24_. Figure 6 C shows the relation between current water storage capacity and the capillary water (mm) in the soil. The capillary water in the soil enhances with increasing trend in the current water storage capacity (*S*_a_). The figure depicts the stronger positive linear relation among *S*_a_ and capillary water in the soil, and the *R*^2^ value displays that this model explains 91 percent data. Figure 6 D shows the linear relationship between carbon (%) and water drop penetration time (*WDPT*_1) and the coefficient value describes the negative relationship among carbon and *WDPT*_1, which means that the water drop penetration time in fresh soil samples decreased with increasing in carbon content. Also, this model describes only 9 percent of the data. The linear regression between carbon (%) and water drop penetration time (*WDPT*_2) is presented in Fig. 6 E. The relation between dependent and independent variables was slightly positive, which describes that rising carbon (%) value slightly increases the water drop penetration time (*WDPT*_2) up to some extent. Further, the statistical differences among carbon and *WDPT*_2 are significant.

### Principal component analysis

**Fig. 7:**
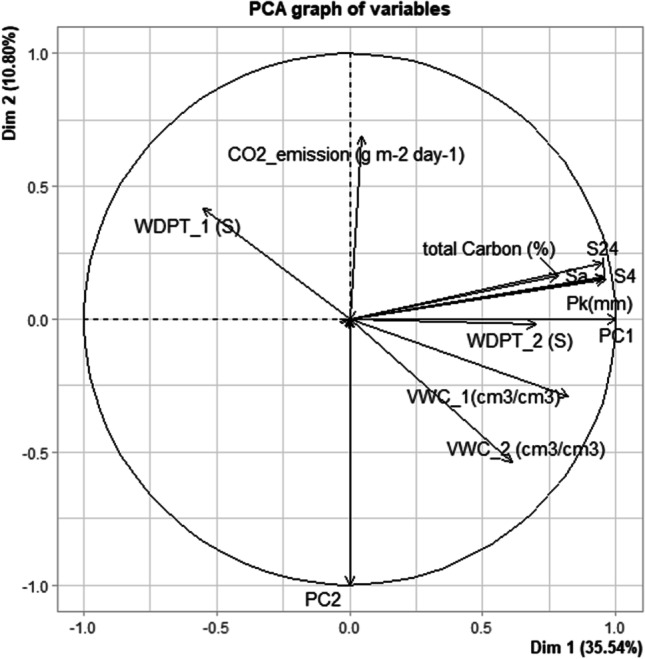
Principal component analysis (PCA) shows variance of the different variables (soil chemical and hydrological properties) of soil measured. *S*_a_, current water storage capacity; *S*_4_, water storage capacity after 4 h; *S*_24_, water storage capacity after 24 h; *VWC*, volumetric water content; *WDPT*, water drop penetration time; *P*_k_, capillary water

A principal component analysis (Fig. 7) was done to evaluate how different doses of SCGs, salt, and sand affect the parameters, such as *S*_a_, *S*_4_, *S*_24_, *VWC*_1 (cm^3^/cm^3^), *WDPT*_1 (S), *VWC*_2 (cm^3^/cm^3^), *WDPT*_2 (S), C (%), CO_2_ (g/m^2^/day), and *P*_k_ (mm). The first two PCA depicted 46.34% total variations among various variables. The CO_2_ emission (g m^−2^ day^−1^), total carbon (%), *S*_a_, *S*_4_, *S*_24_, and *P*_k_ (mm) occupied the upper right quadrant of the plot, and the *VWC*_1, *VWC*_2, and *WDPT*_2 occupied the lower right quadrant. The PCA graph shows the strong relationship of total carbon (%) with *S*_a_, *S*_4_, *S*_24_, and *P*_k_ (mm); however, the relationship between the total carbon (%) with *VWC*_1, *VWC*_2, and *WDPT*_2 was very weak. Additionally, the *VWC*_1 (cm^3^/cm^3^) and *WDPT*_1 (S) lie in opposite direction to each other. The relationship between total carbon (%) and CO_2_ emission (g m^−2^ day^−1^) was negligible.

## Discussion

### Soil chemical properties

The impact of SCG application to the soil considerably reduced the soil pH by 7 and 14%, respectively. The reduction of soil pH was due to the acidic nature of the spent coffee grounds which have the ability to reduce soil pH. Another possible reason in reduction of soil pH could be due to the organic acids present in the SCGs such as chlorogenic acid and citric acid, which can decrease the soil pH. Hardgrove and Livesley (2016) explained that the application of spent coffee ground increases the soil pH in glasshouse trail, whereas the SCG decreases the soil pH in field trail. Another study conducted by Kasongo et al. (2011) depicted that SCG amendment significantly increased the soil pH. Soil EC (mS/cm) were significantly increased by salt treatment (1 and 2 t ha^−1^) by 143 and 283%, correspondingly. Soil salinity increased due to available soluble salt ions by the application of salt. According to Fay and Shi (2012), increased salinity of roadside soils has been linked to the prolonged usage of salt for winter road maintenance. The total acidity was increased with the application of salt treatment, followed by SCGs. Salt increased total acidity up to 20 and 15% by the application of 1 and 2 t ha^−1^. Salts like Cl^−^ are easily dissolved in moist soil, and this process releases H^+^ into the soil solution, increasing soil acidity. The soil nitrogen (%) was higher in the control plot as compared to various amendment applications, which described that the application of the SCGs to the soil has reduced the nitrogen percentage in the soil. When significant amounts of carbon are introduced to the soil, generally followed the degradation and death of surrounding plants, nitrogen levels in the soil are lowered. The nitrogen that is available to the plant will be rapidly depleted by microorganisms as they break down the new carbon source. Hardgrove and Livesley (2016) described two mechanisms by which spent coffee ground could hinder plant growth, which include biological nitrogen immobilization and phytotoxicity. Another study (Cruz and Marques 2015) suggested that SCGs had no significant effect on the soil nitrogen concentration over time. The addition of SCGs at low level (10%) can be effective in the soil, but the concentration of nitrogen decreased with increasing level (20%) of SCG application to the soil (Cruz et al. 2012). A more recent investigation found that the soil nitrogen content for growing lettuce has significantly decreased (35% reduction with 15% SCG) (Cervera-Mata et al. 2019).

Plants that preferentially take up nitrate or have high N needs should have the greatest growth inhibition due to poor soil NO_3_^−^ availability and net NO_3_^−^ immobilization after SCG soil addition (Kahmen et al. 2009). The amount of carbon (%) in the soil has significantly influenced by the various treatments. The highest carbon (%) content was achieved at the rate of 2 t ha^−1^ SCGs followed by SCG (1 t ha^−1^) amendment. Comino et al. (2020) investigated the impact of SCG on two types of soils for a shorter period of 30 and 60 days and concluded that 2.5 and 10% SCG application showed enhancement in the organic matter fraction of the soil. Hirooka et al. (2022) described that the application of SCGs had no significant effect on total carbon and nitrogen content in soil after first year of application, while the top-dressing application of SCGs after 2nd and 3rd year had significantly enhanced the soil total carbon and nitrogen concentration. The CO_2_ emission was not significantly impacted by applying various treatments (SCGs, salt, and sand) at various levels (1 and 2 t ha^−1^). SCGs reduced the CO_2_ emission as compared to other treatments due to C sequestration in the soil. Abagandura et al. (2019) concluded that the addition of biochar and manure reduced CO_2_ emission in the sandy soil.

### Soil hydrological properties

The volumetric water content (*VWC*_1) in the fresh soil samples was increased by SCGs (2 t ha^−1^). The reason behind the highest *VWC*_1 was the organic nature of the SCGs which enhances the physical structure (soil bulk density, specific surface area, soil structure, and total porosity) of the soil, thus increased volumetric water content. The volumetric water content (*VWC*_2) in soil samples after 5 days was retained by SCG application (1 and 2 t ha^−1^) up to 20 and 21%, which showed that soil can retain water for more time even in drought condition could be due to the presence of soil organic matter up to certain limit. When compared to the control soils, the proportion of applied soil water that percolated through the soil columns was considerably (*p* < 0.05) lower for the amended soils, demonstrating an improvement in water retention capacity (Kasongo et al. 2011). Cervera-Mata et al. (2023) concluded that the amount of water retention at field capacity and permanent wilting point was increased with increasing amount of SCGs. The lowest water drop penetration time (*WDPT*_1) in the fresh soil samples was recorded in the SCG treatment (2 t ha^−1^), while the highest time for water drop penetration time (*WDPT*_2) after 5 days was also noted in soil sample taken from the plot treated with (SCGs, 2 t ha^−1^). The more time taken by the drops to absorb in the soil could be due to the presence of organic matter in the SCGs which release some hydrophobic compounds due to which water repelled by the soil surface. Due to an increase in the hydrophobic nature of organic matter in soil in dry conditions, organo-mineral coatings could reduce the wettability of aggregate surfaces (Vogelmann et al. 2013). Fu et al. (2021) concluded that SOC content was positively associated with the persistence of soil water repellency. They also explained that wettable soils had SOC contents of less than 2%, and water-repellent soils had SOC contents of more than 4%. Studies that focused on a particular land-use and soil types indicated positive associations among *WDPT* and soil organic matter in air-dried soils (Lozano et al. 2013; Martínez-Zavala and Jordán-López 2009), but other research indicated weak relationships when multiple land uses were considered (Doerr et al. 2006, 2009). The current water storage capacity (*S*_a_), water capacity after 4 h (*S*_4_), and water capacity after 24 h (*S*_24_) were significantly enhanced by the incorporation of the SCGs (2 t ha^−1^) as compared to other treatments. The enhancement of the *S*_a_, *S*_4_, and *S*_24_ may be due to the organic amendments which improve soil physical properties (soil structure, soil porosity, soil texture, and soil bulk density). Several soil characteristics have been shown to improve after organic waste amendment, including soil water- and nutrient-holding capacity, soil structure and water infiltration, and long-term carbon sequestration (Haider et al. 2014; Diacono and Montemurro 2010; Quilty and Cattle 2011). SCGs can be applied as mulch or as a soil additive. Similar to other mulch materials, it reduces soil temperature when used as mulch and keeps water in the soil by having a high water-holding capacity (Ballesteros et al. 2014). Adi and Noor (2009) asserted that the fine grinding of SCGs offered a number of benefits, including improving the texture of the compost and increasing its water retention capacity. Ndede et al. (2022) reported the same result that the sand-biochar combination underwent aggregation after being thoroughly mixed, which strengthened its physical structure and increased its capacity to hold more water for a longer period of time. Hardgrove and Livesley (2016) resulted that the incorporation of spent coffee grounds at 5% considerably enhanced water-holding capacity of sandy and loamy soil in glasshouse experiment. They proposed that the application of spent coffee grounds at the rate of 20% had considerably resulted in better moisture content than those compared with spent coffee grounds (10%) can lead to soil hydrological benefits. The mixing of organic matter can quickly raise the WHC of less water holding capacity soils (Basso et al., 2013). The capillary water in the soil was enhanced notably after the amendment of the SCGs (1 and 2 t ha^−1^) as compared to other treatments. The presence of organic matter in the soil treated with SCGs might improve the soil porosity and soil structure resulted in high capillary water availability.

The linear regression between current water storage capacity (*S*_a_) and water capacity after (4 and 24 h) depicted that increasing current water storage capacity also increases the water storage capacity for 4 and 24 h. Consequently, the current available water storage directly affects the soil’s water storage capacity after 4 and 24 h. Also, the linear regression between current water storage capacity (*S*_a_) and water capillary in the upper layer of soil was strongly correlated with each other. As the current water storage (*S*_a_) in the soil increases, the capillary water in the soil also increases. The linear regression between carbon (%) and water drop penetration time in fresh and dry samples showed negative and slightly positive correlations, which explains that increasing carbon content in the soil decreases and increases water drop penetration time in fresh and dry soil samples. The reason behind increasing water drop penetration time in dry sample may be because the organic matter may release some hydrophobic compounds which make the soil water repellent. Organic hydrophobic or amphiphilic chemicals that are coated on mineral surfaces or in the interstitial space are what create the phenomenon of SWR (Doerr et al. 2000). The presence of SWR can be influenced by the following factors: vegetation type, microbial activity, soil texture, soil *VWC*, and chemical properties of OM (Doerr et al. 2000). Similar results were shown by Fu et al. (2021) that revealed the soil water repellency persistence characteristics (*WDPT*, *θ*_low_, and *θ*_non_) were significantly linked with soil organic carbon concentration, indicating that soil organic carbon plays a crucial role in the evolution of soil water repellency. According to earlier research (Jeyakumar et al. 2014; Deurer et al. 2011; Lozano et al. 2013), SWRP is likely to increase with a rise in SOC. The likelihood of this occurred because soils with higher SOC contents typically contained more hydrophobic organic components (Mao et al. 2016). Studies that focused on a particular land-use and soil types indicated positive associations among water drop penetration time and soil organic carbon in air-dried soils (Lozano et al. 2013; Martínez-Zavala and Jordán-López 2009), but other research indicated weak relationships when several land uses were considered (Doerr et al. 2006, 2009).

## Conclusions

The obtained results from this experiment concluded the following conclusions:

1. The addition of SCGs (2 t ha^−1^) to the soil decreased the CO_2_ emission from the soil as compared to the control treatment, which suggested that carbon was stored in the soil as a result of carbon sequestration. Therefore, it is suggested to use organic matter in the urban soil in order to mitigate greenhouse gases emission from the soil.
2. The addition of SCGs enhanced the hydrological properties of the soil, which are essential for lower vegetation and tree growth in urban areas. The salt application in the urban soil during the wintertime enhances the soil salinity to a great extent, which destroys the growth of small vegetation and trees near roads. As a result, the urban greenery is deteriorating due to the extensive use of salt on the roads in the wintertime. To combat the detrimental effects of salt, it is advisable to utilize organic material in urban soil, such as SCGs, compost, farmyard manure, or biochar.
3. The single-dose application of different soil amendments had not changed soil chemical and hydrological properties very well. Therefore, it is suggested to conduct future research with SCGs converted to biochar, or SCGs combined with farmyard manure, compost, or biochar with more than one dose of application.

## Data Availability

Data is available from the corresponding author with a formal request.
